# Temporality of heparin-induced antibodies: a retrospective study in outpatients undergoing hemodialysis on unfractionated heparin

**DOI:** 10.1186/s40164-018-0115-8

**Published:** 2018-09-14

**Authors:** Satish Maharaj, Simone Chang, Karan Seegobin, James Morales, Agnes Aysola, Fauzia Rana, Marwan Shaikh

**Affiliations:** 1 Division of Hematology and Medical Oncology, Department of Internal Medicine, University of Florida College of Medicine-Jacksonville, UF Health Jacksonville, 653 W 8th St, Jacksonville, FL 32209 USA; 2 University of Miami Health System, Miami, FL USA

**Keywords:** Antibodies, Hemodialysis, Heparin, HIT, Thrombocytopenia, Thrombosis

## Abstract

**Background:**

Heparin-induced antibodies (HIA) are responsible for causing heparin-induced thrombocytopenia and thrombosis. Research has shown that the temporality of heparin-induced antibodies does not follow the classic immunologic response. The immunobiology of HIA generation remains unclear with varying in vitro and in vivo data. Outpatients undergoing hemodialysis (HD) are exposed to heparin chronically. The HIA immune response can therefore be investigated in vivo in this population.

**Methods:**

We examined the time between the start of HD using unfractionated heparin and HIA levels in 212 outpatients during a 6-year period. Antibodies were detected on enzyme-linked immunosorbent assay. HIA levels were analyzed to determine significance of the trend over time. HIA subgroups were also analyzed for correlation with subsequent thrombotic events and platelet count during follow up.

**Results:**

Overall, the HIA response in HD was found to peak early with waning antibody response despite continued exposure to heparin. The peak prevalence of a strong immune response (optical density > 1.000) was early and short lived, while weaker immune response (optical density 0.400–1.000) persisted for the first 6 months then declined. The mean follow-up time per patient was 2.3 ± 1.4 years. Despite circulating HIA, including high titers, no patients developed HIT in this sample. There was no association between HIA and thrombocytopenia. There was increased incidence of thrombosis in patients with strong HIA compared to other groups, but this did not achieve statistical significance.

**Conclusions:**

The data suggest a significant temporal pattern of HIA in outpatients undergoing HD using unfractionated heparin. Positive HIA was not found to be significantly associated with thrombocytopenia or thrombosis risk in these patients. However, while not achieving statistical significance, subsequent thrombotic events occurred most frequently in the strong positive HIA group (optical density > 1.000). Further research into HIA and risk of thrombosis in this population is needed.

## Background

With the advent of novel therapies, heparin is one of few medications to have endured the test of time, and recently crossed 100 years of use. This year marks 60 years from the first report of heparin-induced thrombocytopenia (HIT) [1]. Heparin is unique, having the highest negative charge density of any known biological molecule. Related in part to this, in a small percentage of patients it paradoxically induces an intense prothrombotic state (HIT). Over the past two decades there has been an explosion of research into the antibody response causing HIT. Our current understanding of HIT is an atypical misdirected antibacterial host defense mechanism involving platelets, monocytes, endothelial cells, innate and adaptive immunity [2–4].

Fundamental to this discovery was recognizing the temporality of heparin-induced antibodies (HIA) does not match the classic immunologic response [5]. HIT is a clinical syndrome defined by HIA reactive to platelet factor 4 (PF4) and heparin complexes. We now know that some individuals have detectable HIA but remain asymptomatic, without thrombocytopenia or thrombosis [2, 6]. The immunobiology and significance of HIA remain perplexing. Heparin exposure has been linked to HIA formation in the orthopedic and cardiac surgery populations [7]. Warkentin et al. [7] reported HIA prevalence of 14.1% and 50% in these populations respectively, but with an unexpected dissociation between HIA and risk for thrombocytopenia and thrombosis. In another cardiac cohort, prevalence after surgery was 26% and found to persist for 3 months [8]. In the cardiac population, HIA without thrombocytopenia have been reported to be a predictor of myocardial re-infarction and thrombotic events [8, 9], although this is debated [10].

Medical outpatients undergoing hemodialysis (HD) are similarly exposed to heparin. However, they go on to have repeated exposure, in many cases lifelong. In the United States, unfractionated heparin (UFH), which is associated with a tenfold higher risk of HIT than low molecular heparin (LMWH), is the most commonly used HD anticoagulant. Over the last two decades there have been several studies investigating HIA prevalence and significance in this population, with varying, and at times, conflicting results. While the prevalence of HIT in HD is very low (< 1%) [9, 11], the prevalence of HIA has been reported to range from insignificant to widely prevalent. The clinical significance of HIA is also debated.

Defining temporal patterns helps not just in clinical application, but also in providing insight into HIA immunobiology. This knowledge will have direct applicability for HIA/HIT testing in patients on HD. Patients on HD continue to increase, with an estimated half-million in the US [12]. Beyond patients on HD, there may be implications for other populations chronically exposed to heparin. Therefore, we set out to investigate HIA trends over time in outpatients on HD and also their clinical significance.

## Methods

This was a retrospective study done using a subpopulation of the outpatient hemodialysis clinic at the University of Florida during the 6-year period from January 1, 2012 to December 31, 2017. This clinic is part of an academic health system serving an urban population. Data collection and analysis was preceded by approval from the university’s project registry, reference number 437. We examined HIA testing in 212 patients over the age of 18 years with end-stage renal disease receiving outpatient HD. All patients received anticoagulation at the start of and during HD using UFH. There were 300 enzyme-linked immunosorbent assays (EIA) performed during this time. This was a retrospective analysis and tests were done purely for surveillance by the nephrologist (no clinical suspicion of HIT or reaction to heparin), without any defined protocol on collection, and no intervention was performed as a result of testing. The timing of testing was therefore determined retrospectively and sampling was random. Testing was done through the HD clinic either at the post-HD initiation visit (102 samples within 1 month) or at a routine follow up visit in the clinic (111 samples over the next 11 months; 87 samples after the first 12 months). Some patients (n = 88) received testing twice, once at initiation and another at a subsequent visit.

Demographic and baseline data were collected on the date of EIA testing. Ethnicity was classified as African-American, Caucasian, or other (Asian-American, Hispanic, and other minority groups). Duration of hemodialysis was derived from physician documentation and defined as the difference between date of hemodialysis initiation and date of EIA testing, approximated to the nearest week. Follow-up time was defined as the time from EIA testing to the end of the study period or death (four mortalities were recorded). Otherwise, there was no loss to follow-up in the sample, with a mean follow-up time per patient of 2.3 ± 1.4 years. History of thrombosis was defined as arterial or venous thromboembolic events (AVTE) occurring prior to EIA testing, and any subsequent AVTE was recorded. Specifically, HD access graft thrombosis was noted. This was defined based on patient symptoms and/or HD flow limitation, followed by positive diagnostic imaging (ultrasonography or angiography) of the access site. Platelet counts were obtained from routine monthly phlebotomy in the clinic.

Testing was done using a commercially available standardized solid phase EIA kit to detect HIA (IgG, IgA and IgM) directed against platelet factor 4 (PF-4) complexed with polyvinylsulfonate (Genetic Testing Institute, Wisconsin). In accordance with specifications, serum was incubated at room temperature in duplicate with reagent and optical density (OD) was measured at 405 nm using reference filter of 490 nm. Quality control was done with positive control OD ≥ 1.800 and negative control at OD ≤ 0.300. OD on duplicate testing was required to fall within 20% of the mean of the two values, or serum was re-tested. The heparin neutralization procedure (HNP) was done on positive samples (OD > 0.400) by adding excess heparin (10,000 U/mL) to the patient and control samples, bringing them to a concentration of 100 U/mL, and the incubation and measurement of OD done as before. Inhibition of a positive reaction by 50% or more in the presence of excess heparin increases specificity for HIA implicated in platelet activation. All assays were performed by experienced staff in the central hospital laboratory. EIA results were recorded in optical density (OD) units and HNP in percentage.

For categorical analysis, data were classified into five HIA groups; defined as HIA negative (OD < 0.400), HIA weak positive (OD 0.400–1.000), HIA strong positive (OD ≥ 1.000) or HIA positive (OD ≥ 0.400) and HNP > 50%. The Χ^2^ test was used to compare categorical variables for independence. The t-test was used for continuous variables. For variance the f-test and analysis of variance (ANOVA) were used. The p-value was set at 0.05 for significance.

## Results

### Patient characteristics

Table 1 shows sample demographics overall and compared by HIA category. Overall, the majority of the sample was African-American and middle-aged, with both sexes balanced. Female sex is known to be associated with a higher risk of HIT [13]. The only significantly different subpopulation was the HIA strong positive group, where patients were more likely (p < 0.05) to be Caucasian and male.

**Table 1:** Demographics and patient characteristics, comparing mean values by categorical HIA status

	Overall	HIA negative (OD < 0.400)	HIA positive (OD ≥ 0.400)	HIA positive and HNP > 50%	HIA strong positive (OD ≥ 1.000)
Age (years; mean ± SD)	54 ± 15	52 ± 14	55 ± 15	56 ± 20	64 ± 13
Female sex (%)	53	53	52	55	30*
Body mass index (kg/m^2^; mean ± SD)	28.8 ± 8.2	32 ± 8.3	28.5 ± 7.7	27.8 ± 8.3	27.4 ± 5.1
Time on dialysis (months; mean ± SD)	16.3 ± 29.1	17.8 ± 27.3	12.5 ± 33.0	13.3 ± 36.4	8.3 ± 25.1
Ever smoker (%)	33.3	35.1	29.2	24.2	18.2*
History of cancer (%)	9.7	9.2	7.1	3.1*	9.1
Race (%)					
African American	81	81	81	76	72*
Caucasian	13	13	12	12	28*
Other	6	6	7	12	0*

* *p *< 0.05 for independence

### Overall HIA prevalence and distribution

**Fig. 1:**
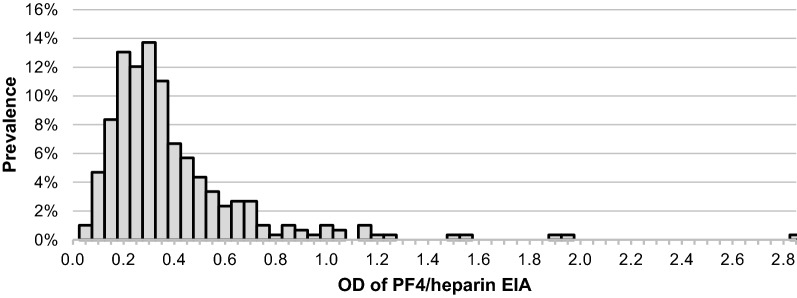
Distribution of HIA in chronic hemodialysis using unfractionated heparin. The antibody responses demonstrate continuous unimodal distribution skewed to the right with high OD outliers

Heparin-induced antibodies were detected in 32.5% (69/212) of the sample using the EIA threshold of OD ≥ 0.400. When a positive EIA was combined with positive HNP testing (OD ≥ 0.400 and HNP > 50%) prevalence was 15.6%. The prevalence of high titer HIA (OD ≥ 1.000 and HNP > 50%) was 5.2%. In Fig. 1 the OD frequencies for the entire sample are charted. In this sample of HD patients the EIA threshold for positivity (OD = 0.400) occurred just above the mode. There was a clear unimodal distribution, with a rightward skew and high OD outliers. The data indicates in this population there was a continuum of the immune response resulting in variable anti-PF4/heparin antibody production.

### Temporality of HIA in hemodialysis

**Fig. 2:**
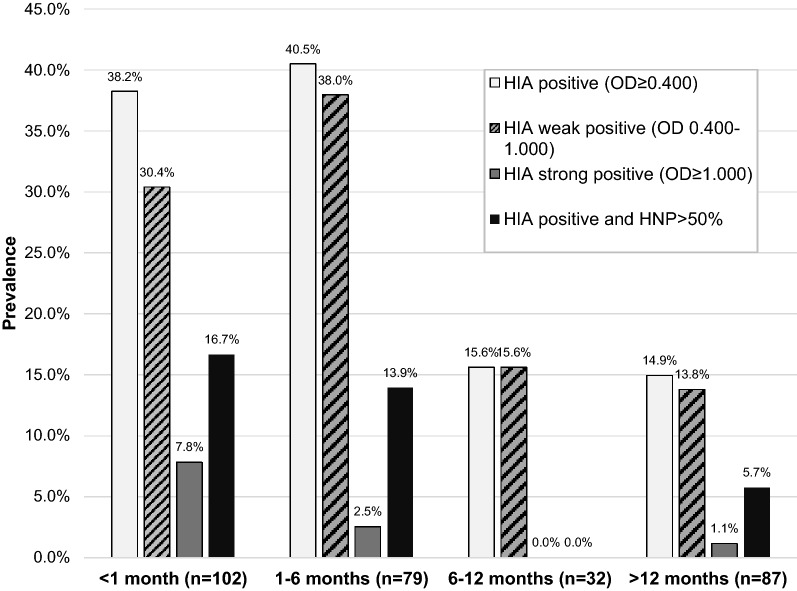
Temporality of positive HIA in hemodialysis using UFH. Anti-PF4/heparin antibodies are produced along a continuum, but there is waning of the immune response with increased time on hemodialysis. The strongest antibody generation is within the first 6 months

**Fig. 3:**
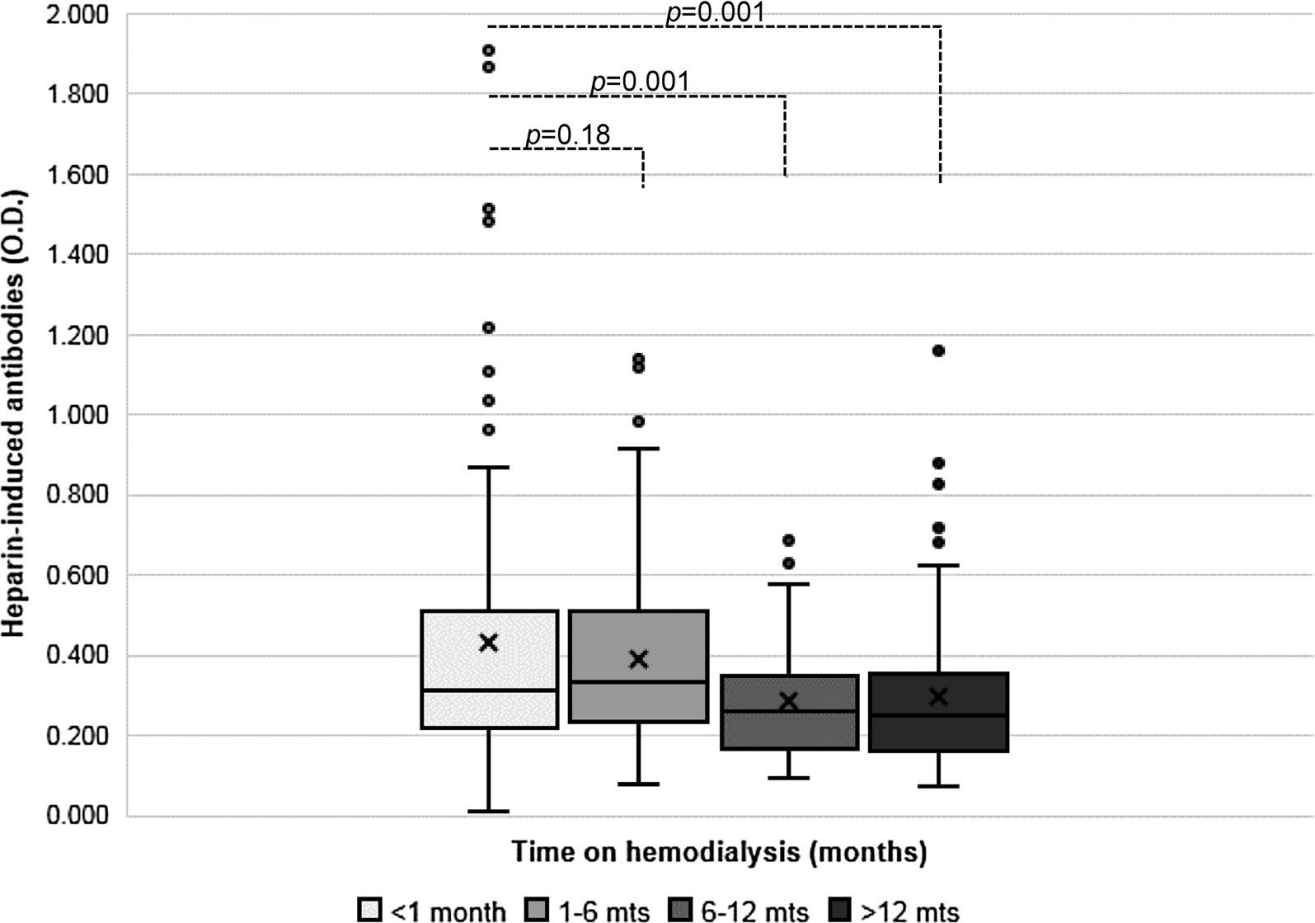
Plot of all HIA levels (OD) stratified by time and compared for significance. The box plots show the 25th percentile to the 75th percentile, with the midline representing the median and separating the two quartiles. The first whisker (below) shows the lowest 25%; while the second whisker (upper) shows the top 25%, excluding outliers. The mean OD is denoted by the “X” marker. Outliers are designated by values greater than 1.5 times the interquartile range. A significant decline in OD was noted after 6 months

To determine the temporality of anti-PF4/heparin antibodies, HIA levels (OD) were examined according to time on HD. A categorical analysis is presented in Fig. 2. The prevalence of a strong immune response (OD ≥ 1.000) was early, peaking at < 1 month with a rapid decline right after. Patients with weak immune response (OD 0.400–1.000) also had a very early response but this persisted longer with peak prevalence during the first 6 months. Patients who developed more specific antibodies (OD ≥ 0.400 and HNP > 50%) likewise demonstrated early peak prevalence with gradual decline and persistence during the first 6 months. For both weak and strong antibody responses, there was a decline after reaching a maximum, in all groups, despite the continued use of UFH. Figure 3 shows the entire spread of HIA levels (OD) stratified by time. There is a spectrum of HIA production at any given time period, but at 6 months there is significant waning of the immune response (p = 0.001) despite continued UFH exposure. Within the first 6 months on hemodialysis there is the most robust immune response.

### HIA and platelet count

When compared among time matched groups, HIA levels did not correlate significantly with mean platelet count (Table 2; p > 0.05 for independence among all groups). There was no clear trend in mean platelet count based on HIA status. As discussed above, HIA titers were highest during the first 6 months, and during this time there was no association with thrombocytopenia in any group. Therefore, we did not find evidence that HIA are associated with thrombocytopenia in outpatients on chronic HD.

**Table 2:** Mean platelet counts (×1000/µL; mean ± SD) categorized by HIA and time

Time on hemodialysis	HIA negative (OD < 0.400)	HIA positive (OD ≥ 0.400)	HIA weak positive (OD 0.400–1.000)	HIA strong positive (OD ≥ 1.000)
≤ 1 month	250 ± 103	281 ± 158	282 ± 170	277 ± 101
1–6 months	246 ± 87	219 ± 133	218 ± 169	239 ± 130
6–12 months	219 ± 99	183 ± 131	182 ± 133	–
> 12 months	166 ± 78	136 ± 129	135 ± 130	149*
All	215 ± 95	232 ± 129	228 ± 131	258 ± 117

* n = 1

### HIA and thrombotic events, including access thrombosis

The prevalence of AVTE and access thrombosis were assessed at baseline and throughout follow up and are presented in Table 3. To address sample heterogeneity, the distributions of classical thrombosis risk factors are also presented. Risk factors analyzed were previous thrombosis, cancer, known thrombophilia (inherited or acquired), obesity and major surgery or pregnancy during the follow up time period. Prevalence of smoking is already shown in Table 1. All of these thrombosis risk factors were analyzed for significance to determine potential confounding, but none achieved statistical significance (p > 0.05). Next, subsequent AVTE and vascular access thrombosis were analyzed between HIA negative and positive groups. While the prevalence of subsequent AVTE and access thrombosis were highest in the HIA strong positive group, neither this nor any of the other groups was found to be statistically significant (p > 0.05).

**Table 3:** Prevalence of thrombosis risk factors at baseline and thrombotic events during follow up, classed according to HIA status

	Overall (%)	HIA negative (OD < 0.400) (%)	HIA positive (OD ≥ 0.400) (%)	HIA weak positive (OD 0.400–1.000) (%)	HIA positive and HNP > 50% (%)	HIA strong positive (OD ≥ 1.000) (%)
History of AVTE (baseline)^a^	12.75	14.10	10.00	8.50	12.12	18.20
History of cancer	8.51	9.20	7.10	6.80	3.03	9.10
Hypercoagulable state^b^	8.44	9.10	7.10	8.50	6.10	0.00
Obesity (BMI > 30 kg/m^2^)	54.67	58.40	47.10	47.50	39.40	45.40
Major surgery or pregnancy during follow up	1.87	2.10	1.40	1.70	0.00	0.00
Subsequent AVTE	32.07	32.40	31.40	30.50	30.30	36.40
Subsequent vascular access thrombosis^c^	18.87	19.00	18.60	16.90	12.12	27.30

^a^Including vascular access occlusion (1 event in a HIA negative case), provoked and unprovoked events

^b^Known diagnosis of inherited or acquired thrombophilia (antiphospholipid syndrome, essential thrombocythemia, polycythemia vera, protein C/S deficiency); HbSS; amyloidosis; chronic use of medications associated with thrombosis; chronic inflammatory states

^c^Excluding central venous catheter thrombosis

## Discussion

In patients exposed to unfractionated heparin through HD, HIA were found to have a temporal pattern. The peak prevalence of a strong immune response (OD ≥ 1.000) was early and short lived. There is a spectrum of HIA production at any given time period, but the first 6 months on hemodialysis generate the most robust immune response. From 6 months onward there is significant waning of the immune response. The data suggest that HD patients who mount a robust HIA response do not maintain this over time despite continued heparin exposure.

It has been shown that during recovery from HIT, there is a decline in antibody response and the average case becomes HIA negative at 3 months [14]. In HIT patients this was initially thought to be from heparin cessation, but Greinacher and colleagues [5] also observed a decline of antibody reactivity on EIA despite continued exposure to UFH at prophylactic doses in orthopedic and trauma patients. The waning antibody response despite continued exposure to heparin contrasts with typical IgG mediated drug reactions, in which strong antibody response persists for years after exposure. This pattern supports the theory that there is active down-regulation of the HIA immune response and induction of peripheral tolerance mechanisms [15].

The waning immune response found raises the question of T-cell involvement in HIA production. T-cell involvement generates protective memory with immune recall which is absent in our data as well as other studies into HIT. Nevertheless, in a murine immunization model, athymic mice lacking T-cells did not exhibit HIA response which was generated by wild type mice [16]. Similarly, in another murine model, inducing T-helper cell depletion with anti-CD4 antibody significantly impaired the HIA response [17]. Greinacher and others [5, 18] have proposed the HIA immune response is T-cell independent, mediated by B-cells. In one hypothesis, the antigenic complexes present repetitive PF4 tetrameric epitopes similar to those in viruses that cause T-cell independent B-cell reactivity [5]. It has also been recently found that PF4-heparin complexes activate complement and bind to B-cells via complement receptors (CD21) [19]. Further research is required to bridge murine and clinical studies. It may be that peripheral tolerance mechanisms are regulating B and T-cell crosstalk [20, 21].

Over the last two decades, a wide range of HIA prevalence in HD has been reported in studies using EIA, ranging from 0 to 47% [22–37, 39]. The finding that HIA levels inversely correlate with time on HD helps explain the wide variance as these studies did not standardize time on HD. When thirteen of these studies were combined in a review, a total HIA prevalence of 8.1% for patients on UFH and 1.8% for LMWH was found [38]. We found a prevalence higher than this. However, many of the studies at that time were small and there were differing EIA (OD) thresholds and varying times on HD. More recently, using the same EIA as this study, an analysis of the German Diabetes Dialysis Study (n = 1236) found an HIA prevalence of 18.7% [39]. Interestingly, periodontal disease has been correlated with HIA [40] and there is a higher prevalence of periodontitis in the HD population [41, 42]. It should also be noted that African-Americans are under-represented in published data [34].

The diagnosis of HIT requires laboratory confirmation that is usually done first by EIA. When compared to functional assay, EIA is less costly, more widely available, rapid, reproducible, and does not involve radiation. However, from this data and others, the high prevalence of HIA in patients undergoing HD using UFH decreases accuracy of EIA in the diagnosis of HIT, particularly in the first 6 months after initiating HD. Indiscriminate EIA testing in HD patients will undoubtedly result in HIT overdiagnosis, thus unnecessarily exposing patients to alternative anticoagulation. The HD population has a higher chance of HIT overdiagnosis on EIA and serology should not be ordered when clinical suspicion is low. An OD of 0.400 is conventionally used as the cutoff for “positive” and “negative” on EIA. It has been shown in some populations that increasing OD threshold enhances specificity without compromising sensitivity. This would be worthwhile to study in chronic HD. In these patients we emphasize that interpreting the actual OD value in the clinical context is much more useful than a qualitative approach.

Finally, this study confirms the clinical heterogeneity of HIA. The presence of circulating HIA has been associated with rapid-onset HIT [14] and most providers would hesitate to administer heparin to such cases. However, in this sample, continued exposure to heparin did not result in HIT or significant adverse thrombotic outcomes. Despite circulating HIA, including high titers, no patients developed HIT with continued exposure to heparin. There was no significant association between HIA and thrombocytopenia, AVTE or access thrombosis. It has been suggested that asymptomatic but detectable HIA contributes to hypercoagulability and access thrombosis in HD [27, 30, 43]. However, neither our study nor others [26, 32] strongly corroborate this finding. This deserves further investigation.

Recently, Nazi and colleagues [44] investigated a modified serotonin release assay (SRA) able to detect platelet-activating antibodies below the threshold of the standard SRA. They found that 36% of EIA positive but SRA negative samples had low levels of platelet-activating HIA. Therefore, they suggested HIA can be platelet-activating or non-platelet-activating, and a critical threshold must be crossed to initiate clinical HIT. Additional mechanisms, host and immunologic factors, result in HIA becoming pathogenic in some while others remain asymptomatic. Patient-specific differences in B-cell binding of PF4/heparin/complement complexes may be involved [19, 20].

Our study has several limitations. This was a single center study and the population studied was limited in size, predominantly urban and African-American. We acknowledge that retrospective studies such as this are susceptible to heterogeneity. Nevertheless, the demographics (Table 1) and data spread (Fig. 1) do not suggest significant heterogeneity in the population, except for ethnicity where African-Americans comprise majority of the sample. For statistical analysis the distribution and variances were assessed in determining significance. The analysis of thrombophilia was limited to already known diagnoses and systematic screening of all patients was not done (such as Protein C and S levels) so it is possible some patients had higher thrombosis risk than known. We reported on access thrombosis in this study as a factor of interest. Some patients in this study were on antiplatelet or anticoagulant therapy. We did attempt to assess the usage of these therapies, but the rates of non-adherence noted were so high that this data was not reliable. This factor can potentially affect the rates of graft thrombosis.

## Conclusions

The data suggest a significant temporal pattern of HIA in outpatients undergoing HD with continued exposure to unfractionated heparin. Positive HIA was not found to be significantly associated with thrombocytopenia or thrombosis risk in these patients. However, while not achieving statistical significance, subsequent thrombotic events occurred most frequently in the strong positive HIA group (OD > 1.000). The atypical immune response to heparin and clinical heterogeneity of HIA continue to be intriguing. Further research into HIA and risk of thrombosis with in vitro and in vivo studies in this population is needed.

## Abbreviations

AVTE: arterial or venous thromboembolic events
EIA: enzyme-linked immunosorbent assay
HIA: heparin-induced antibodies
HD: hemodialysis
HIT: heparin-induced thrombocytopenia
HNP: heparin neutralization procedure
LMWH: low molecular heparin
OD: optical density
SRA: serotonin release assay
UFH: unfractionated heparin
